# Hyperelastic Ex Vivo Cervical Tissue Mechanical Characterization

**DOI:** 10.3390/s20164362

**Published:** 2020-08-05

**Authors:** Antonio Callejas, Juan Melchor, Inas H. Faris, Guillermo Rus

**Affiliations:** 1 Department of Structural Mechanics, University of Granada, 18010 Granada, Spain; inas@ugr.es (I.H.F.); grus@ugr.es (G.R.); 2 Instituto de Investigación Biosanitaria, ibs.GRANADA, 18012 Granada, Spain; jmelchor@ugr.es; 3 Excellence Research Unit, “Modelling Nature” (MNat), University of Granada, 18010 Granada, Spain; 4 Department of Statistics and Operations Research, University of Granada, 18010 Granada, Spain

**Keywords:** hyperelasticity, uniaxial tensile test, cervical tissue, nonlinearity, hyperelastic models

## Abstract

This paper presents the results of the comparison between a proposed Fourth Order Elastic Constants (FOECs) nonlinear model defined in the sense of Landau’s theory, and the two most contrasted hyperelastic models in the literature, Mooney–Rivlin, and Ogden models. A mechanical testing protocol is developed to investigate the large-strain response of ex vivo cervical tissue samples in uniaxial tension in its two principal anatomical locations, the epithelial and connective layers. The final aim of this work is to compare the reconstructed shear modulus of the epithelial and connective layers of cervical tissue. According to the obtained results, the nonlinear parameter *A* from the proposed FOEC model could be an important biomarker in cervical tissue diagnosis. In addition, the calculated shear modulus depended on the anatomical location of the cervical tissue ($\mu_{epithelial}$ = 1.29 ± 0.15 MPa, and $\mu_{connective}$ = 3.60 ± 0.63 MPa).

## 1. Introduction

Modeling of soft tissue implies new perspectives that carry several clinical applications. It could be used, for example, in tissue engineering [1,2,3], for finite element modeling [4,5,6,7], to analyze virtual reality in clinical practice [8,9] and for surgery planning [10,11]. To simulate those applications, the theory of linear elasticity has been employed to understand the results of mechanical tests on soft tissues [12,13]. However, surgical procedures lead to consider large displacements and linear elasticity is a simplification when considering small strains. There is a need among researchers to use simplified models which can represent the nonlinear behavior of soft tissues. The simplicity of the proposed model in conjunction with a good correlation with the experimental data can be presented as an accurate and simple model in computational solid mechanics field.

Although the nature of soft tissue behaviour is viscoelastic [14], a simplification of hyperelasticity allows a reasonable characterization of the mechanical properties, specifically when the loss of strain energy is small (low loading rates). Veronda and Westmann [15] and Fung [16] were the first works that used hyperelasticity for soft tissue modeling. The hyperelastic approach postulates the existence of the strain energy function which relates the displacement of the tissue to the corresponding stress values [17]. The most common strain energy functions for the modeling of soft tissues are polynomial forms, such as Mooney–Rivlin and Ogden models. Many authors have modeled the behaviour of soft tissues such as, porcine spleen, porcine kidney, porcine liver, rat or human brain [18,19,20,21,22]. Regarding cervical tissue, uniaxial tension tests [23,24,25,26,27] and compression [25,28,29] have been studied in rat tissue and human tissue using load-relaxation protocols. A nonlinear stress–strain response has been shown in the tension and compression tests and the response of the tissue was noticeably stiffer in tension than in compression. It was observed that tissue from pregnant patients was one to two orders of magnitude more compliant than tissue from nonpregnant patients [25,29]. In a work carried out by Yoshida et al. [27], load relaxation ring tests were performed on pregnant and nonpregnant rat cervices. The pregnant tissue showed a very large stress-relaxation compared to the nonpregnant tissue. Myers et al. observed that the cervix stiffness changes along its length in the uniaxial tensile test, where the external os had a stiffer response than the internal os [25]. The relationship between stiffness and gestational age was studied by Poellmann et al. and Jayyosi et al. [26,30]. The works concluded that stiffness decreased as gestational age increased. In the works mentioned above were uniaxial, compression and traction tests were performed, the mechanical properties of the tissues have been obtained. However, in those works the nonlinear elastic properties of ex vivo human cervical tissue, using the Fourth Order Elastic Constants (FOECs), Ogden, and Mooney-Rivlin models have not been obtained through uniaxial tensile tests yet.

Soft tissues are composed of several layers; each one of these layers has different compositions, for instance, cervical tissues have an epithelial outer layer and a connective layer. The connective layer is composed by an extracellular matrix (ECM) that ensures the strength and integrity of the cervix, resisting shear deformation, through a fibrous scaffold [31]. The main component of the ECM is fibrillar collagen, which determines a cross-linked network interlaced with the elastin protein, enclosed by a ground substance of proteoglycans and glycosaminoglycans [32,33,34]. Researchers have identified three zones of structured collagen in the connective layer: the innermost and outermost rings of stroma contain collagen fibers preferentially aligned in the longitudinal direction, and the middle layer contains collagen fibers preferentially aligned in the circumferential direction [35,36]. Regarding the collagen content, the middle zone had higher levels of collagen content when compared with the inner and the outer zones [36]. According to the mechanical studies on soft tissues, the connective layer is often considered as the most important from a mechanical point of view [37,38,39]. However, other studies, based on Torsional Wave Elastography, consider the epithelial layer as a key apart from the connective one [40,41,42]. The reason is that torsional waves not only propagate in depth but along the surface before being registered by the receiver. One of the purposes of this work is to study the differences in stiffness between the epithelial and connective layers of ex vivo human cervical tissue that comes from the hyperelastic models employed.

The main aim of this work is to propose a new hyperelastic model based on FOEC in the sense of Landau’s theory. The evaluation of the agreement between classical hyperelasticity theory and acoustoelasticity theory, in particular third and fourth order elastic constants, in predicting the mechanical response has potentially high impact. Recent advances in medical imaging techniques for non-invasive evaluation of rheological behavior of soft tissues provides new perspectives, in particular for in vivo quantification of nonlinear shear wave-based elastographic biomarkers, which could be diagnostic predictors of a broad spectrum of labor disorders.

This work has been divided into several sections. First, a brief introduction to the core content has been presented. Second, a section of materials and methodology details the methods used in this work. Uniaxial tensile tests of human ex vivo cervical tissues were performed to fit the hyperelastic models. Third, the results of the comparison between the experimental test and the hyperelastic models were analyzed.

## 2. Materials and Methods

### 2.1. Theory of Hyperelastic Models

This section shows the theoretical relationship between stress and strain for a proposed hyperelastic model based on the FOEC in the sense of Landau’s theory, Mooney–Rivlin and Ogden models.

#### 2.1.1. Proposed Fourth Order Elastic Constants Nonlinear Model

Nonlinear FOECs are defined in the sense of Landau’s theory [43] to establish a strain energy function, considering the medium incompressible valid for the hyperelastic regime as defined Hamilton and Destrade [44,45],

(1) $$W=\mu I_{2}+\frac{1}{3}AI_{3}+DI_{2}^{2}$$

where $I_{1}=\mathrm{tr}E,I_{2}=\mathrm{tr}E^{2}$ and $I_{3}=\mathrm{tr}E^{3}$ are the classical invariant of deformation defined by Cemal et al. [46], E is the Green strain tensor, μ is the shear modulus and A and D are the Third and Fourth Order Elastic Constants of Landau respectively. The Second Piola–Kirchoff stress tensor is determined by a constitutive law as follows,

(2) $$S=\frac{\partial W}{\partial E}$$

where E is the Green–Cauchy strain tensor defined in terms of displacement field as the difference between actual and initial position respectively, u=x−X. This strain tensor is defined, according to the large deformation theory, as,

(3) $$E=\frac{1}{2}\left({\left(\nabla_{X}u\right)}^{T}+\nabla_{X}u+{\left(\nabla_{X}u\right)}^{T}+\nabla_{X}u\right)$$

**Figure 1:**
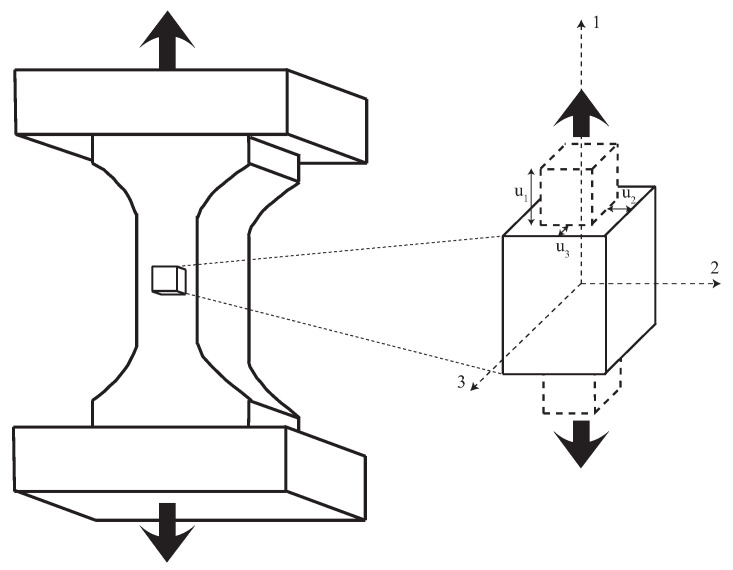
(**Left**): scheme of the uniaxial tensile test. (**Right**): zoom of a differential element of the sample.

Under the hypothesis of a tensile test setup, the initial conditions are described in Figure 1. where the displacements are defined in three directions as,

(4) $$\begin{array}{c} u_{1}=ax_{1}\\ u_{2}=-bx_{2}\\ u_{3}=-bx_{3} \end{array}$$

In this case, the Green–Cauchy strain tensor defined in Equation (3) may be described in matrix form as,

(5) $$E=\left(\begin{array}{ccc} a+\frac{1}{2}a^{2} & 0 & 0\\ 0 & -b+\frac{1}{2}b^{2} & 0\\ 0 & 0 & -b+\frac{1}{2}b^{2} \end{array}\right)$$

To describe the Second Piola–Kirchoff stress tensor in a nonlinear regime, it is necessary to determine the invariant $I_{3}$ in terms of strains.

(6) $$\begin{array}{c} I_{3}=E_{11}^{3}+E_{22}^{3}+E_{33}^{3}\\ \frac{\partial I_{3}}{\partial E}=\left(\begin{array}{ccc} 3E_{11}^{2} & 0 & 0\\ 0 & 3E_{22}^{2} & 0\\ 0 & 0 & 3E_{33}^{2} \end{array}\right) \end{array}$$

The constitutive law for tensile test case in direction 1 is deduced by the expression,

(7) $$S_{11}=2\mu a+\left(\mu +A\right)a^{2}+\left(A+4D\right)a^{3}$$

The relationship between the Cauchy stress tensor and the Second Piola–Kirchoff stress tensor is defined as,

(8) $$\sigma =J^{-1}FSF^{T}$$

where F is the deformation gradient tensor and J=det(F).

The derivation of Cauchy stress tensor in the context of weakly nonlinear elasticity [47] yields the constitutive law defined in high order as follows,

(9) $$\sigma_{11}=2\mu a+\left(5\mu +A\right)a^{2}+\left(7\mu +3A+4D\right)a^{3}+\left(\frac{5}{2}\mu +3A+8D\right)a^{4}+\frac{5}{2}\left(A+4D\right)a^{5}$$

In order to compare with the other two hyperelastic models, the aforementioned tensor is simplified (using μ and *A*) as follows:

(10) $$\sigma_{\mathrm{NL}}=2\mu a+\left(5\mu +A\right)a^{2}$$

where *a* is defined in Equation (4).

#### 2.1.2. Mooney-Rivlin Model

The Mooney–Rivlin model, originally derived by Mooney in 1940 [48] was formulated in terms of the Cauchy–Green deformation tensor invariants by Rivlin [49] as:

(11) $$\Psi =\sum_{i=1}^{2}c_{i}\left(I_{i}-3\right)$$

where $c_{1}$ and $c_{2}$ are the material parameters, $I_{1}$ and $I_{2}$ the first and second strain invariants respectively and Ψ the strain energy function.

In the case of an uniaxial tension ($\sigma =\sigma_{1}$, $\sigma_{2}=\sigma_{3}=0$) the Cauchy stress as a function of the strain invariants is

(12) $$\sigma =2\left(\lambda^{2}-\frac{1}{\lambda }\right)\left(\frac{\partial \Psi }{\partial I_{1}}+\frac{1}{\lambda }\frac{\partial \Psi }{\partial I_{2}}\right)$$

where $\lambda =\lambda_{1}$ ($\lambda_{1}$ is the principal stretch in 1 direction) and the invariants from the Cauchy–Green tensor for an incompressible hyperelastic material subjected to a uniaxial tension are defined as [50],

(13) $$\begin{array}{cc} I_{1} & =\lambda^{2}+\frac{2}{\lambda }\\ I_{2} & =2\lambda +\frac{1}{\lambda^{2}}\\ I_{3} & =1 \end{array}$$

For the Mooney–Rivlin model, the Cauchy stress obtained employing (12) and using two parameters ($c_{1}$ and $c_{2}$) is,

(14) $$\sigma_{\;\mathrm{Mooney}}=2\left(\lambda^{2}-\frac{1}{\lambda }\right)\left(c_{1}+c_{2}\frac{1}{\lambda }\right)$$

#### 2.1.3. Ogden Model

The strain energy function in the Ogden model, developed in 1972 [51], is described by,

(15) $$\Psi =\sum_{r=1}^{N}\frac{\mu_{r}}{\alpha_{r}}\left(\lambda_{1}^{\alpha_{r}}+\lambda_{2}^{\alpha_{r}}+\lambda_{3}^{\alpha_{r}}-3\right)$$

where $\mu_{r}$ (infinitesimal shear modulus) and $\alpha_{r}$ (stiffening parameter) are material constants, and $\lambda_{1}$, $\lambda_{2}$ and $\lambda_{3}$ are the principal stretches. Taking into account that for an incompressible material, $\lambda_{1}=\lambda$ and $\lambda_{2}=\lambda_{3}=1/\sqrt{\lambda }$ [50], Equation (15) is simplified into,

(16) $$\Psi =\sum_{i=1}^{N}\frac{\mu_{r}}{\alpha_{r}}\left[\lambda^{\alpha_{r}}+2{\left(\frac{1}{\sqrt{\lambda }}\right)}^{\alpha_{r}}-3\right]$$

The Cauchy stress tensor as a function of the principal stretches for an incompressible material is,

(17) $$\sigma_{1}=\lambda_{1}\frac{\partial \Psi }{\partial \lambda_{1}}-\lambda_{3}\frac{\partial \Psi }{\partial \lambda_{3}}$$

Finally, using Equation (17), the Cauchy stress using two parameters ($\mu_{r}$ and $\alpha_{r}$) is obtained as follows,

(18) $$\sigma_{\mathrm{Ogden}}=\mu_{r}\left(\lambda^{\alpha_{r}}-\lambda^{-\alpha_{r}/2}\right)$$

The shear modulus μ in the Ogden model results from the expression,

(19) $$\mu =\frac{\mu_{r}\alpha_{r}}{2}$$

### 2.2. Hysterectomy Specimens

A total of seven hysterectomy specimens from women with benign gynecological conditions were obtained from Health Campus Hospital in Granada (Table 1). The study met the principles of the Declaration of Helsinki. Approvals of the Ethical Committee in Human Research of the University of Granada and Ethical Commission and Health Research of Health Campus Hospital in Granada were achieved. All women enrolled in the evaluation provided agreement by signing a written consent and reading the information of the patient report.

### 2.3. Mechanical Tests

**Figure 2:**
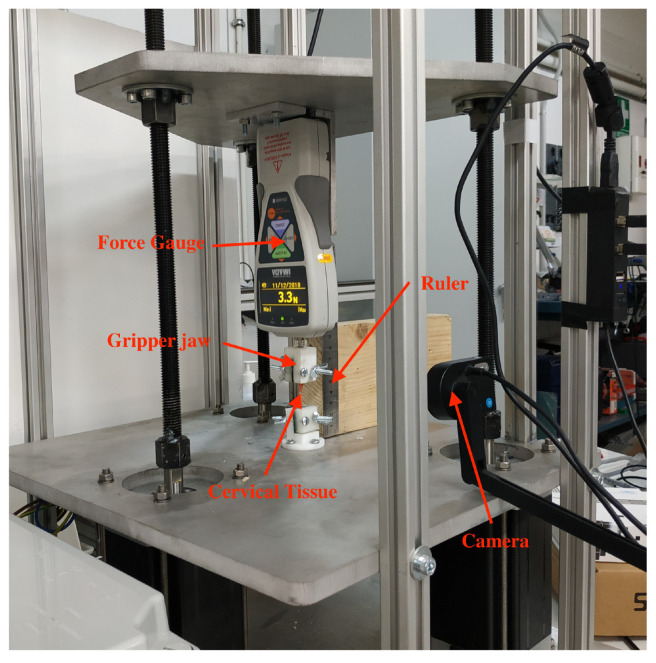
Experimental setup comprising a 500 N force gauge, gripper jaws for holding the sample attached and a conventional camera to register the loading process.

**Figure 3:**
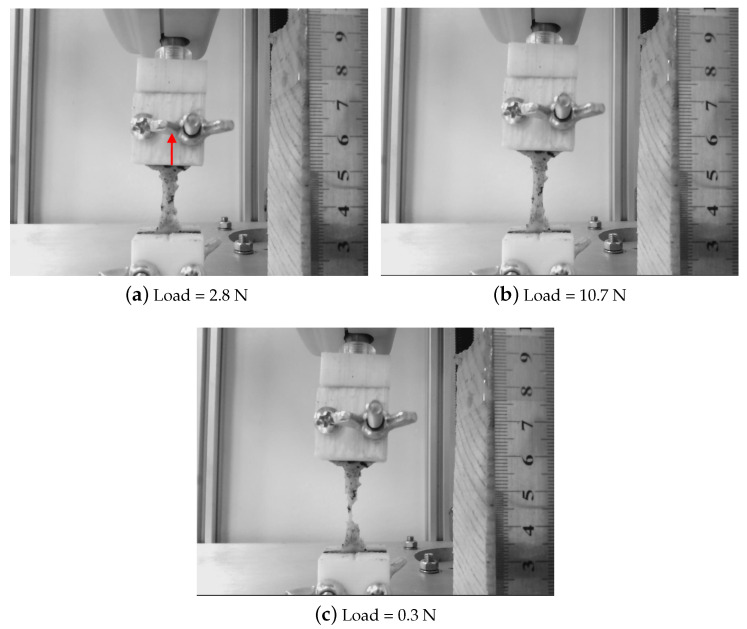
Three different frames from a recording of a uniaxial tensile test in a cervical tissue sample. The tissue is stretched in the direction marked with a red arrow.

All the mechanical tests were performed using the tensile-compression press shown in Figure 2. The device was equipped with a 500 N force gauge (IMADA ZTA-500N) fixed to a platform that is operated by three motors with an accuracy of 0.3 μm. The tolerance of the force gauge is 0.1 N. The cervical tissue was fixed by two Acrylonitrile Butadiene Styrene (ABS) printed gripper jaws, one was attached to the press and another linked to a fixed support, that prevents the cervical tissue from undesired movements. According to the literature reviewed in soft tissue uniaxial tensile tests, the load step was 0.2 mm, and the strain ramp rate used was 1%/s [52]. A rule was used in the same plane in which the sample was contained for the calculation of deformations. Finally, a conventional camera (IPEVO Ziggi-HD High Definition USB CDVU-04IP model, 5 Mpix, 1280 × 720 resolution) was employed to acquire the image sequence at each loading step until the sample breakdown (Figure 3). The camera was synchronized with a MATLAB^®^programming environment (Release 2018b, MathWorks, Natick, MA, USA) at the beginning of the experimental test. The code implemented in MATLAB^®^allowed controlling each increment of load through an Arduino microcontroller, at the same time that recorded at a rate of 1 frame per load increment until the sample breakdown.

**Figure 4:**
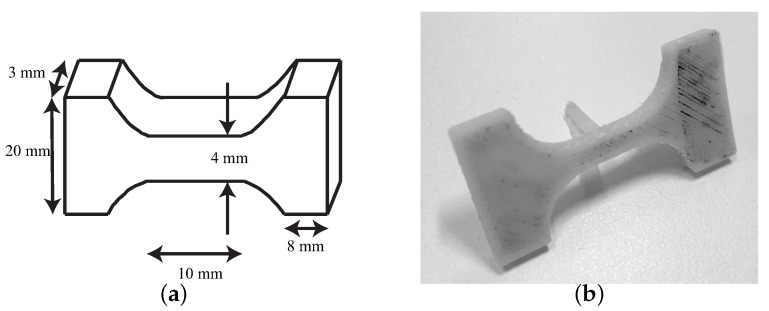
(**a**) Mold printed with Acrylonitrile Butadiene Styrene (ABS) to maintain the geometry of the samples. (**b**) Cervical tissue sample geometry.

**Figure 5:**
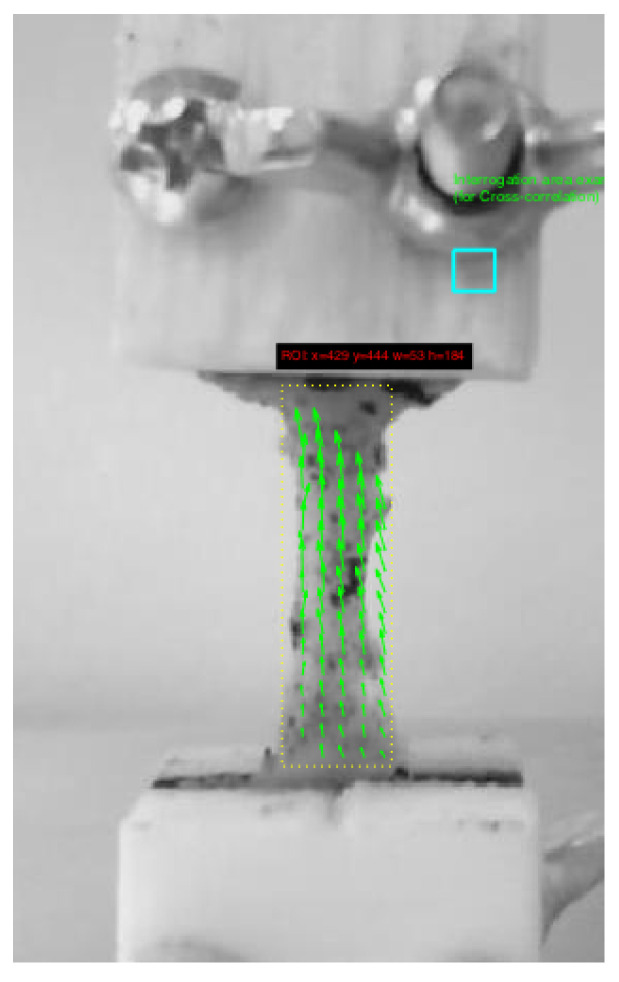
An illustrative example of cervical tissue attached to two gripper jaws that fix it during the uniaxial tensile test. A dashed yellow line was used to delimit the region of interest (ROI). The green arrows represent the displacements.

The sample preparation protocol consists of several steps:

1. All the seven cervical tissues were excised from the women and placed in phosphate buffered saline (PBS) to avoid loss of hydration after surgery. The connective layer was cut below the epithelial layer, and at a sufficient distance from the cervical canal to ensure that the preferred direction of the collagen fibers corresponds to the direction of the uniaxial tensile test [29,53]. The samples were tested in the Ultrasonics Laboratory at the University of Granada. Two slices were cut manually from each cervical sample, one from the epithelial layer and another one from the connective layer. The epithelial layer was cut carefully to obtain a thickness between 0.5 and 1 mm. The connective layer was obtained below the epithelial layer. All the samples were cut with the same mold (see Figure 4) to maintain the same geometry, which is necessary to locate the most unfavorable section.
2. A random dot pattern was used in the cervix to improve deformation monitoring carried out by a cross-correlation algorithm (PTVlab software), see Figure 5. For the speckle generation, acrylic black paint was used.
3. An optimal contrast obtained by a good illumination and a uniform background help the tracking algorithm.
4. It is worth underlining that the cervical tissue samples were kept continuously hydrated so as not to alter the mechanical properties during the experiment by spraying them with PBS.
5. All the samples were preconditioned with 10 cycles at 1 N before the uniaxial tensile test.

PTVlab is free software that was developed by Dr. Wernher Brevis (mainly developed the mathematical algorithms) and Antoine Patalano (an adaptation of the graphical user interface (GUI) in MATLAB and the development of new functionalities) [54,55]. The Large Scale Particle Tracking Velocimetry (LSPTV) method is employed by PTVlab and uses the binary correlation, the Gaussian mask and the dynamic threshold binarization techniques for the particle detection. A Gaussian mask with a correlation threshold 0.5 and a sigma of 3 px was used for particle tracking. The Particle Tracking Velocimetry (PTV) algorithm was cross-correlated by an interrogation area of 10 px, a minimum correlation of 0.6 px, and a similarity neighbor of 25%. The deformations were calculated in the most unfavorable area of the cervical tissue, which according to the printed mold corresponds to the central area.

## 3. Results

### 3.1. Comparison between Hyperelastic Models

**Figure 6:**
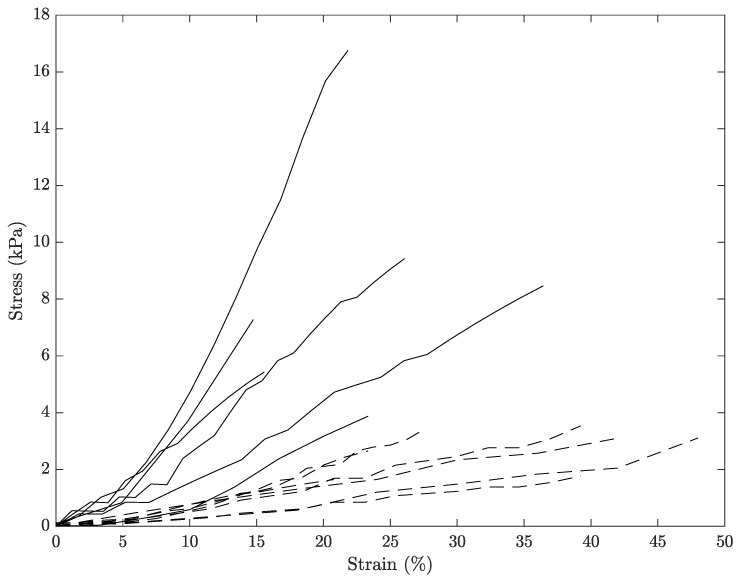
Experimental stress-strain relationship for cervical samples tested under uniaxial tensile test. Solid black and discontinue lines represent the connective and layer respectively. The stress is the true stress.

**Figure 7:**
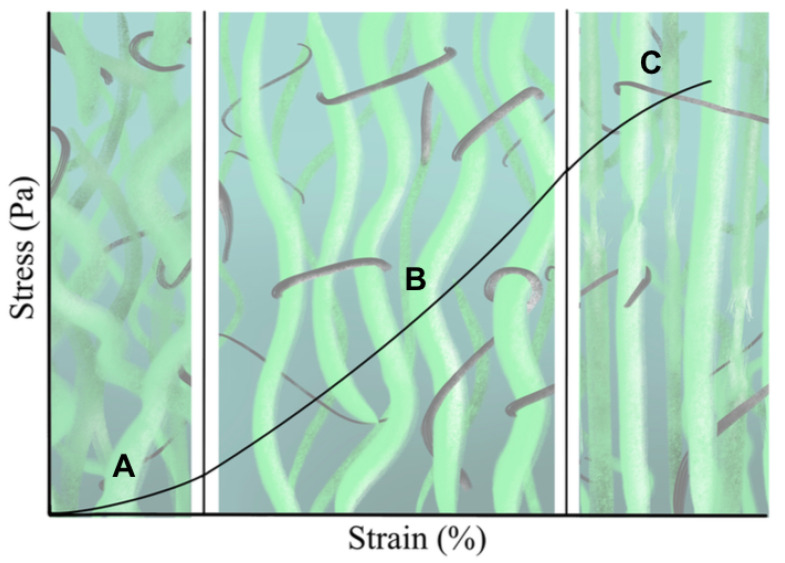
Representation of stress–strain behavior of soft tissues. The curve is divided into three zones: nonlinear (**A**), quasi-linear (**B**) and rupture (**C**). The state of elastin (black color) and collagen (green color) is represented at the bottom of each zone.

**Figure 8:**
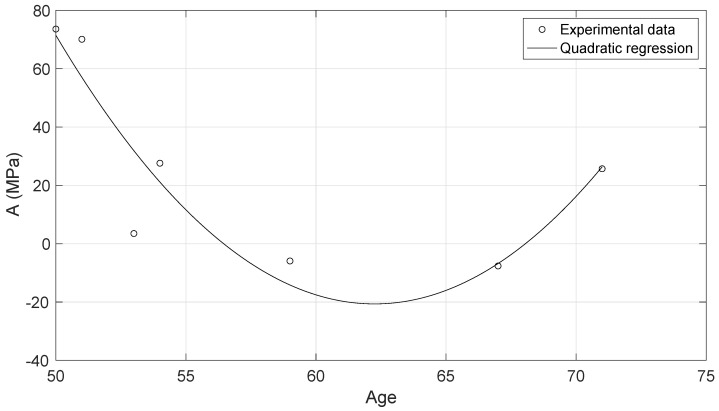
Quadratic regression of the Third Order parameter *A* of the connective layer against the woman’s age. $R^{2}=0.84$.

**Figure 9:**
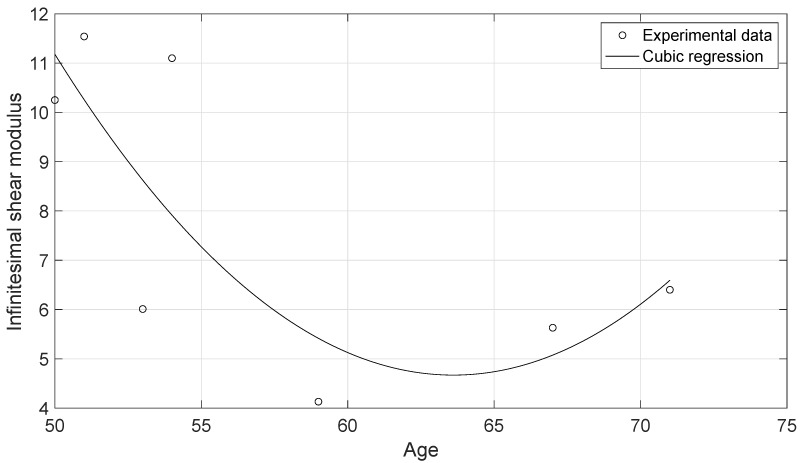
Cubic regression of the infinitesimal shear modulus $\mu_{r}$ of the connective layer from the Odgen model against the woman’s age. $R^{2}=0.60$.

**Figure 10:**
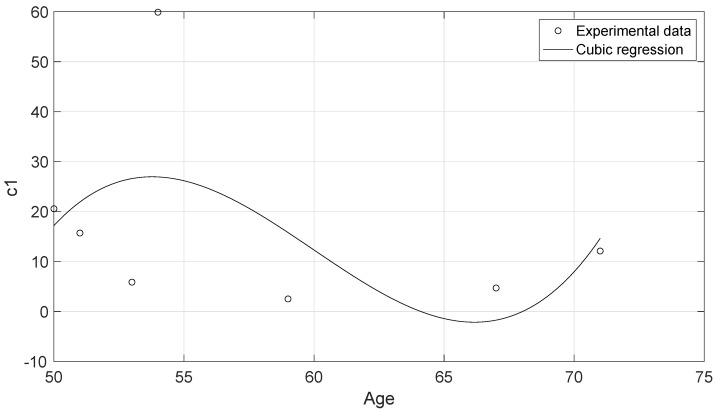
Cubic regression of the $c_{1}$ parameter of the connective layer from the Mooney–Rivlin model against the woman’s age. $R^{2}=0.24$.

**Figure 11:**
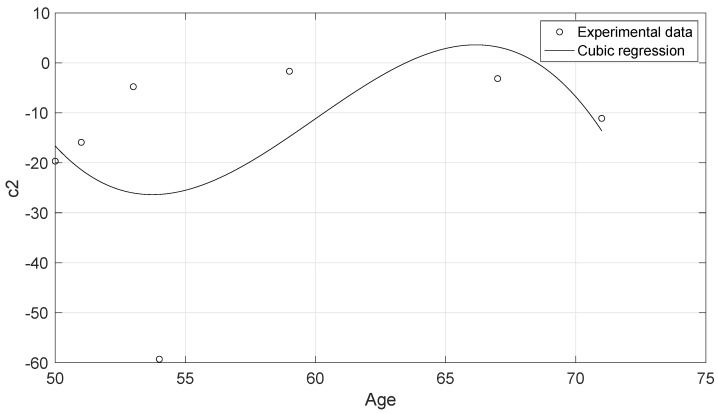
Cubic regression of the $c_{2}$ parameter of the connective layer from the Mooney–Rivlin model against the woman’s age. $R^{2}=0.25$.

The experimental data of the uniaxial tensile tests for each of the cervical tissue samples are represented as stress–strain curves (Figure 6). In these curves, it can be appreciated the three zones that are explained in Figure 7: nonlinear, quasi-linear and rupture. The results of the fits of the experimental data with the three hyperelastic models are shown in Table 2, Table 3 and Table 4. These fitted curves were performed with MATLAB *®* (Release 2018b, MathWorks, Natick, United States) Curve Fitting Toolbox. The median and the confident intervals have been calculated for each parameter. The relationship between woman’s age and the Third Order Elastic Constant *A* from the proposed model, the infinitesimal shear modulus $\mu_{r}$ from the Ogden model, $c_{1}$ parameter from the Mooney–Rivlin model, and $c_{2}$ parameter from the Mooney–Rivlin model for the connective layer are shown in Figure 8, Figure 9, Figure 10 and Figure 11.

**Figure 12:**
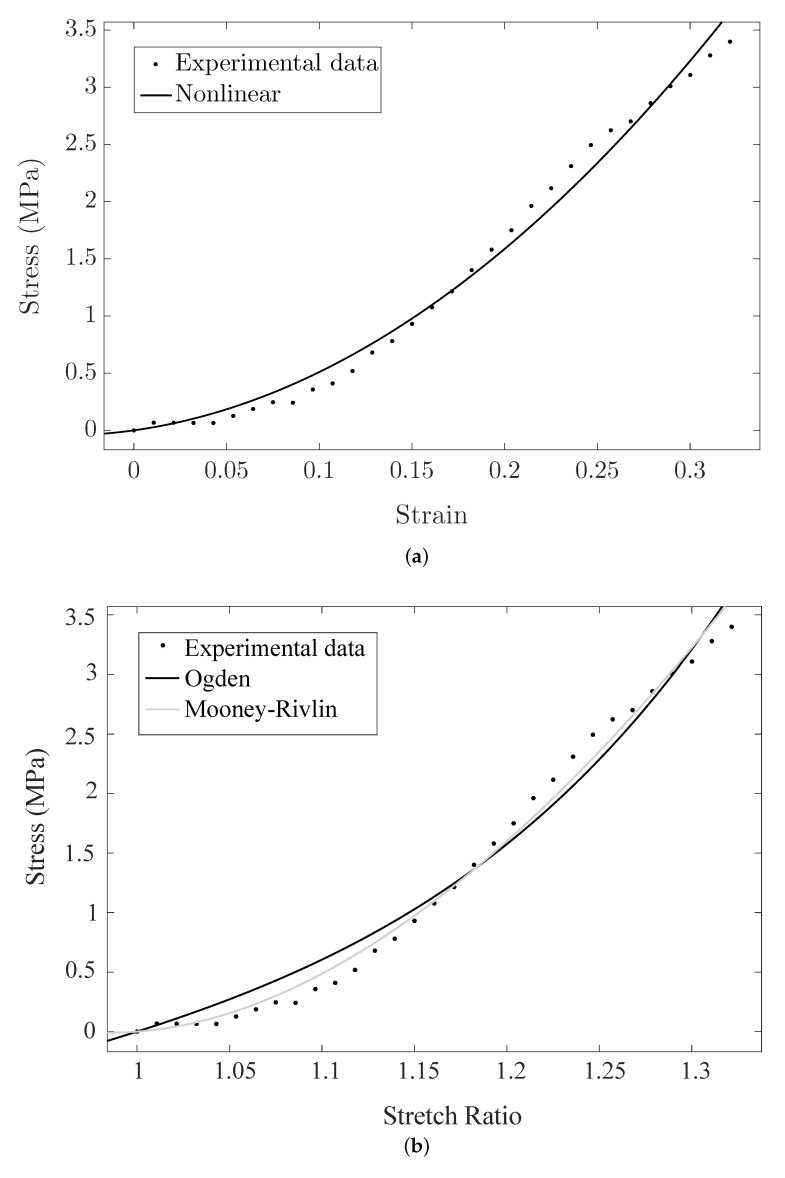
Comparison of the hyperelastic theoretical models with the experimental results obtained from the connective layer of Cervix 2. (**a**) The proposed nonlinear Fourth Order Elastic Constant (FOEC) nonlinear model; (**b**) Mooney–Rivlin and Ogden models.

An illustrative example of the comparison of the hyperelastic theoretical models with the experimental results obtained from the connective layer of Cervix 2 is showed in Figure 12.

### 3.2. Shear Modulus Estimation

The shear modulus can be obtained directly by means of the μ parameter of the FOEC proposed model, through the slope of the stress–strain curve in the linear region or also trough a combination of the two parameters of the Ogden model, the infinitesimal shear modulus $\mu_{r}$ and the stiffening parameter $\alpha_{r}$ (see Equation (19)). Table 5 shows the values of the shear modulus for each procedure and for each sample.

**Figure 13:**
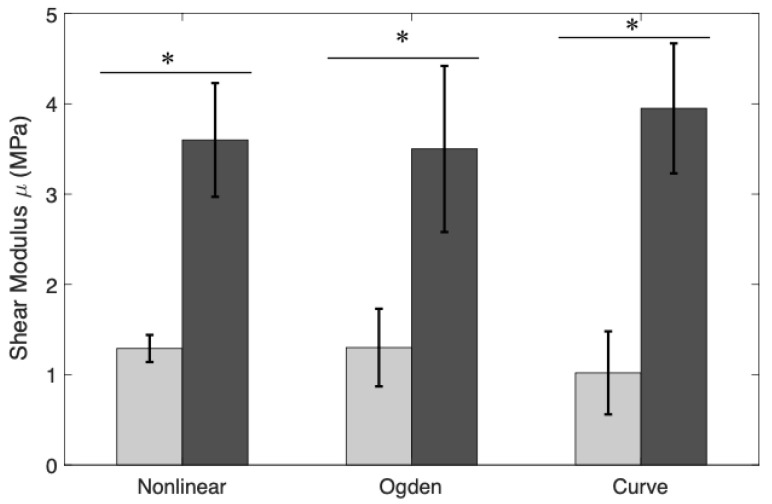
Comparison between shear modulus of epithelial and connective layers using the proposed nonlinear model, the Ogden model, and the slope of the linear region of the stress-strain curve. The results are presented as mean ± standard deviation. The light gray bars represent the epithelial layer and the dark gray bars the connective layer. *p*-value obtained from the Student’s *t*-test was the metric used for this comparison. (* *p*-value < 0.001).

In order to study the differences between the obtained shear modulus with the nonlinear model, Ogden model and the slope of the curve stress-strain for each cervical layer, a Student’s *t*-test was used (see Figure 13).

**Figure 14:**
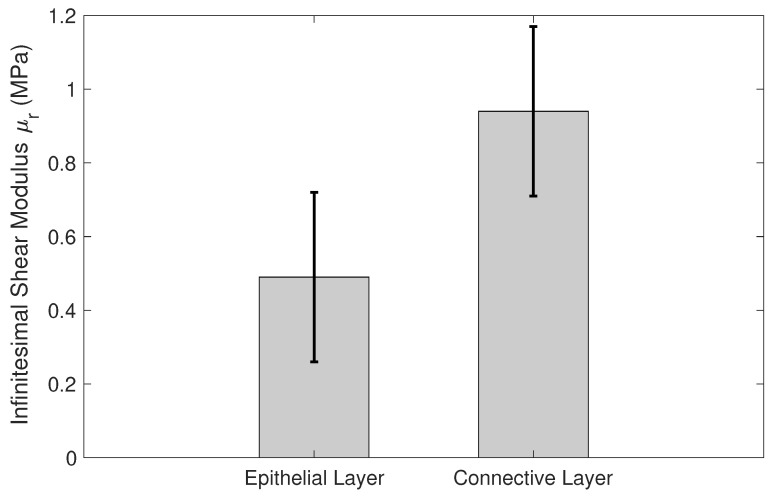
Comparison between the infinitesimal shear modulus ($\mu_{r}$) of epithelial and connective layers using the Ogden model. The results are presented as mean ± standard deviation. *p*-value obtained from the Student’s *t*-test was the metric used. (* *p*-value = 0.0016).

Another parameter that shows significant differences between the epithelial and the connective layers is the infinitesimal shear modulus. Figure 14 shows the mean and deviation values of the infinitesimal stiffness modulus, derived from the Ogden model, for the epithelial and connective layers. The metric used for the comparison was the p-value obtained from the Student’s *t*-test.

## 4. Discussion

According to the evidence found in literature, Myers et al. [29] investigated the nonlinear time-dependent stress response of cervical samples from different human hysterectomy specimens. Results showed the nonlinear response of cervical stroma, which was dependent on obstetric history. However, to our knowledge, the nonlinear elastic properties of ex vivo human cervical tissue, using the aforementioned hyperelastic models, have not been obtained by uniaxial tensile tests yet.

This work aims at representing a first step toward a nonlinear characterization of human cervical tissue. The nonlinear elastic properties of ex vivo cervical tissue have been obtained for the first time by uniaxial tensile tests.

The first contribution of this study is to propose a new hyperelastic model (nonlinear model) based on the FOEC in the sense of Landau’s theory [56] and compare the obtained results with the most used hyperelastic models in the literature, Mooney–Rivlin, and Ogden models [49,51].

As a second contribution, the differences in shear modulus between epithelial and connective layers of ex vivo human cervical tissue are analyzed. The TWE technique proposed in the literature aims to locally measure the mechanical parameters of the cervix that can be correlated with the different stages of the cervix maturing during pregnancy [41]. These waves interact not only with the superficial layer, the epithelial layer, but also with the deeper layers, i.e., connective layer. Therefore, a validation of the mechanical parameters reconstructed in both layers is necessary through uniaxial tensile tests of ex vivo samples from hysterectomies of healthy women.

The mechanical behavior of the cervix is nonlinear, as are most of the soft biological tissues. The nonlinear parameters of the FOEC proposed model were obtained through a fit of the experimental data measured in the uniaxial tensile tests with the theoretical law that governs the hyperelastic model. The same procedure was carried out in order to get the nonlinear parameters from the two most employed hyperelastic models to characterize soft biological tissue in the literature, Mooney–Rivlin and Ogden models.

The proposed FOEC hyperelastic model, Equation (9), presents three parameters, the shear modulus μ, the third order elastic constant *A* and the fourth order elastic constant *D*. In this study, the proposed theoretical stress-strain relationship has been simplified with the aim of comparing with the two-term Mooney–Rivlin and Ogden models. The two parameters that have adjusted the experimental data were the shear modulus μ and the third order elastic constant *A*. Analyzing the results of the shear modulus, there was no significant variation in both, the epithelial 1.29 ± 0.15 MPa and the connective layer 3.60 ± 0.63 MPa for each of the hysterectomy samples, see Table 5. The values of the shear stiffness are highly dependent on the strain ramp rate used, the larger strain rate, the larger shear modulus [57,58]. The values obtained for cervical tissue agree with those found in the literature [23,29]. However, regarding the nonlinear parameter *A*, large variation was observed, varying the parameter from positive to negative values (see Table 2). Previous studies in the literature have obtained the value of parameter A in tissue-mimicking phantoms and breast tissue [59,60]. Gennisson et al. [59] investigated the nonlinear behavior of quasi-incompressible agar-gelatin-based phantoms using supersonic shear imaging technique. The study concluded that, for different samples of tissue-mimicking phantoms, the value of parameter A varied from a negative value to a positive one. Similar results were obtained by Bernal et al. [60] in breast tissue, parameter A presents high variability due to internal changes in tissue consistency. According to the present study, it is worth pointing out that, to our knowledge, this is the first work that studies the nonlinear parameters of the FOEC hyperelastic model in cervical tissue. The variability found in the third elastic constant parameter *A* could be associated with the heterogeneity of the tissue, like the two previous studies [61], also depends on the particular epidemiological aspect of each patient, as reported by Myers et al. [62]. Despite the variability of parameter A, and the low number of samples, the quadratic regression studied in the connective tissue samples showed a high correlation with women’s age ($R^{2}=0.84$) (Figure 8). On the other hand, the cubic regressions in the connective layer of the infinitesimal shear modulus $\mu_{r}$ from the Ogden model, the $c_{1}$ parameter from the Mooney–Rivlin model, and the $c_{2}$ parameter from the Mooney–Rivlin model against the women’s age have smaller correlations, Figure 9, Figure 10 and Figure 11 ($R^{2}=0.60$, $R^{2}=0.24$, and $R^{2}=0.25$ respectively). This result is very preliminary because only seven samples have been considered, so future studies to explore other relationships with physiological markers require a greater number of patients, despite the difficulty of obtaining ex vivo samples.

On the one hand, the shear modulus is one of the most used parameters in the characterization of soft biological tissues by various techniques. This value can be extracted directly by means of the μ parameter of the FOEC proposed model, through the slope of the stress–strain curve in the linear region or also through a combination of the two parameters of the Ogden model, the infinitesimal shear modulus $\mu_{r}$ and the stiffening parameter $\alpha_{r}$. The values of the shear modulus for each reconstruction technique and for each sample are shown in Table 5. The parameters that govern the Mooney–Rivlin model have no physical sense and, therefore, the shear modulus value can not be extracted from them. The results of the shear modulus for each reconstruction technique and for each cervical layers were compared by using a Student’s *t*-test. *t*-test results are shown in Figure 13, in terms of *p*-values. All of these values were below 0.001, which can be considered to represent a significant difference in the shear modulus between the epithelial and connective layers. In conclusion, the shear modulus was dependent on the anatomical location of the cervical tissue as shown in Table 5 and Figure 13. On the other hand, the infinitesimal shear modulus ($\mu_{r}$) is one of the parameters that governs the hyperelastic Ogden model. The p-value obtained for the comparison between the epithelial and connective layers (*p*-value = 0.0016) shows that there is a significant difference (see Figure 14), therefore, this parameter could also indicate differences in stiffness for both layers.

In general, by the obtained results, it can be concluded that the nonlinear parameter A could be an important biomarker in the characterization of connective cervical tissue. In addition, the calculated shear modulus depended on the anatomical location of the cervical tissue. The protocol of measurements is applicable to other tissues and it is possible to explore a set of new nonlinear measurements from different procedures, for example in vivo measurements in women using ultrasound wave propagation by employing Hamilton’s formulation, one step further from the diagnostic point of view.

## 5. Conclusions

In this work, as a first contribution, we proposed a new hyperelastic model (nonlinear model) based on the Fourth Order Elastic Constants (FOECs) in the sense of Landau’s theory to reconstruct the nonlinear parameters in cervical tissue by fitting the experimental data with this model. The experimetal data were also fitted by the most used hyperelastic models in the literature, Mooney–Rivlin, and Ogden. The nonlinear parameter A from the proposed model could be an important biomarker in connective cervical tissue diagnosis. As a second contribution, a comparison of the shear modulus, extracted from three different procedures, between the epithelial and connective layers of ex vivo cervical tissue was performed. The conclusion is that shear modulus was dependent on anatomical location of the cervical tissue. Despite the difficulties encountered in the characterization of the hyperelastic behaviour of cervical tissue, the proposed nonlinear model should be considered as the basis of more complex constitutive equations. Nevertheless, the nonlinear FOEC model should remain as the starting point in the hyperelastic characterization of the cervical tissue in future studies.

## Figures and Tables

**Table 1:** Obstetric characteristics of the population in the study.

Patient	Age	Hysterectomy Indication
**1**	53	Vaginal prolapse
**2**	67	Subserous myoma
**3**	59	Vaginal prolapse
**4**	54	Cervical prolapse
**5**	50	Cervical prolapse
**6**	51	Cervical prolapse
**7**	71	Cervical prolapse

**Table 2:** Results of the fits of experimental data with the proposed nonlinear model. Shear modulus μ and TOEC *A* in MPa. IQR: Interquartile Range.

	Nonlinear Model			
	Epithelial Layer		Connective Layer	
Cervix	μ	A	μ	A
1	1.13	22.6	3.58	3.49
2	1.22	−6.08	4.72	−7.63
3	1.35	−3.06	2.64	−5.92
4	1.57	28.3	3.30	27.6
5	1.35	−2.35	3.51	73.6
6	1.13	2.32	3.49	70.1
7	1.27	30.72	3.96	25.7
**Median (IQR)**	1.27 (1.13 1.35)	2.32 (−3.06 28.3)	3.51 (3.30 3.96)	25 (−5.92 70.1)

**Table 3:** Results of the fits of the experimental data with the Ogden model. The infinitesimal shear modulus $\mu_{r}$ in MPa. IQR: Interquartile Range.

	Ogden Model			
	Epithelial Layer		Connective Layer	
Cervix	$\mu_{r}$	$\alpha_{r}$	$\mu_{r}$	$\alpha_{r}$
1	0.41	7.94	0.941	6.01
2	1.01	1.62	1.16	5.63
3	0.42	4.54	0.97	4.13
4	0.35	9.94	0.85	11.1
5	0.47	4.31	0.82	10.25
6	0.39	5.27	0.57	11.54
7	0.40	9.05	1.29	6.40
**Median (IQR)**	0.41 (0.39 0.47)	5.27 (4.31 9.05)	0.94 (0.82 1.16)	6.40 (5.63 11.1)

**Table 4:** Results of the fits of the experimental data with the Mooney–Rivlin model. IQR: Interquartile Range.

	Mooney-Rivlin Model			
	Epithelial Layer		Connective Layer	
Cervix	$c_{1}$	$c_{2}$	$c_{1}$	$c_{2}$
1	6.93	−6.73	5.87	−4.77
2	0.33	−0.08	4.7	−3.15
3	1.22	−0.78	2.51	−1.68
4	8.25	−7.84	59.9	−59.3
5	1.47	−1.05	20.56	−19.67
6	2.35	−2.06	15.7	−15.9
7	8.69	−8.44	12.1	−11.1
**Median (IQR)**	2.35 (1.22 8.25)	−2.06 (−7.84 −0.78)	12.10 (4.70 20.56)	−11.1 (−19.67 −3.15)

**Table 5:** Shear modulus estimation for the proposed nonlinear model, the Ogden model and the slope of the linear region of the stress–strain curve. The mean and standard deviation of the values for the seven samples are presented in MPa.

	Shear Modulus					
	Epithelial Layer			Connective Layer		
**Cervix**	**Nonlinear**	**Ogden**	**Curve**	**Nonlinear**	**Ogden**	**Curve**
1	1.13	1.65	0.82	3.58	2.83	4.17
2	1.22	0.82	0.69	4.72	3.28	3.78
3	1.35	0.95	1.43	2.64	2.01	3.62
4	1.57	1.77	1.82	3.30	4.71	3.26
5	1.35	1.02	0.44	3.51	4.22	5.25
6	1.13	1.03	0.90	3.49	3.30	4.42
7	1.27	1.84	1.08	3.96	4.15	3.17
**Mean ± Std**	1.29 ± 0.15	1.30 ± 0.43	1.02 ± 0.46	3.60 ± 0.63	3.50 ± 0.92	3.95 ± 0.72

## Abbreviations

The following abbreviations are used in this manuscript:

FOEC	Fourth order elastic constants (FOEC)
ECM	Extracellular matrix
PBS	Phosphate buffered saline
ABS	Acrylonitrile butadiene styrene
GUI	Graphical user interface
LSPTV	Large scale particle tracking velocimetry
PTV	Particle tracking velocimetry
SSR	Sum of squares of the regression
SST	Total sum of squares
